# Trocar localisation for robot-assisted vitreoretinal surgery

**DOI:** 10.1007/s11548-023-02987-y

**Published:** 2023-06-24

**Authors:** Jeremy Birch, Lyndon Da Cruz, Kawal Rhode, Christos Bergeles

**Affiliations:** 1 School of Biomedical Engineering and Imaging Sciences, King’s College London, Strand, London, WC2R 2LS UK; 2 Moorfields Eye Hospital, 162 City Rd, London, EC1V 2PD UK

**Keywords:** Robotics, Eye-surgery, Trocar, Localisation, ArUco, Aurora

## Abstract

**Purpose:**

Robot-assisted vitreoretinal surgery provides precise and consistent operations on the back of the eye. To perform this safely, knowledge of the surgical instrument’s remote centre of motion (RCM) and the location of the insertion point into the eye (trocar) is required. This enables the robot to align both positions to pivot the instrument about the trocar, thus preventing any damaging lateral forces from being exerted.

**Methods:**

Building on a system developed in previous work, this study presents a trocar localisation method that uses a micro-camera mounted on a vitreoretinal surgical forceps, to track two ArUco markers attached on either side of a trocar. The trocar position is the estimated midpoint between the markers.

**Results:**

Experimental evaluation of the trocar localisation was conducted. Results showed an RMSE of 1.82 mm for the localisation of the markers and an RMSE of 1.24 mm for the trocar localisation.

**Conclusions:**

The proposed camera-based trocar localisation presents reasonable consistency and accuracy and shows improved results compared to other current methods. Optimum accuracy for this application would necessitate a 1.4 mm absolute error margin, which corresponds to the trocar’s radius. The trocar localisation results are successfully found within this margin, yet the marker localisation would require further refinement to ensure consistency of localisation within the error margin. Further work will refine these position estimates and ensure the error stays consistently within this boundary.

## Introduction

Vitreoretinal surgery relates to procedures that deal with the back of the eye, including the retina, macula and vitreous fluid. Indications that are dealt with through vitreoretinal surgery procedures include epiretinal membrane peeling, retinal detachment and complications caused by diabetic retinopathy.

During the procedure, the surgeon creates incisions in the eye at a safe area termed the pars plana. Several access ports, called trocars, are placed in these locations to serve as both strain relief and pivoting points for the inserted instruments that are used to access the back of the eye.

In the past decades, there has been an increase in R &D of robot-assisted vitreoretinal surgery systems, see [1]. These include robots to remove hand tremor [2] or entire robot-assisted surgical systems to perform procedures such as occluded retinal vein cannulation [3]. Allowing robots to assist enables greater precision, hand tremor removal and increased consistency, thus improving patient recovery time [1]. Furthermore, they can surpass human ability, allowing direct targeting of areas of interest, such as the subretinal layers, with new regenerative therapies that otherwise would not be possible.

For the robots to pivot safely and accurately the surgical tool about the trocar, precise knowledge of both the tool’s Remote Centre of Motion (RCM) and the trocar location, must be known [4]. The misalignment of these points will exert harmful lateral forces on the eye, causing irreversible trauma.

With this in mind, an system that can be integrated into vitreoretinal robots has to be developed that accurately estimates both points of interest, relaying them back to the control system. Such a system could also be placed on common surgical instruments for use during manual procedures. This would enable the collection of intraoperative data consisting of the tool and trocar positions. This dataset would be useful for deep learning instrument tracking and automated trocar docking or for surgeon training virtual reality platforms.

This paper proposes the trocar localisation aspect of such a system. This is achieved by utilising a micro-camera attached to the surgical instrument to detect two markers found at either side of the trocar, and thereby interpolate its position. This study is divided into the following sections: related work, methods, experiments and results, discussion and conclusion.

## Related work

Several studies have investigated the use of instrumented trocars to self-locate. To do this, [5] used an integrated camera with ceiling markers, whilst [6] used an IMU and range sensor. Instrumented trocars require a large trocar for sensor integration. Whilst being applicable in laparoscopy, they cannot be scaled down to ophthalmic surgery. Least-squares algorithms have also been used to estimate the intersection point of a surgical instrument pivoting about the trocar. The main assumption of this method is that the pivoting point equates to the trocar. The work in [7] and [8] used the robot kinematics for this, whilst [9] estimated the trocar based on endoscope poses, and [10] used external stereo-cameras to detect the instrument. These algorithms assume that the tool’s pivoting point equates to the trocar position, which does not hold for hollow suspended organs such as the eye, where the two points can be misaligned causing harmful lateral forces. Finally, deep learning networks have been used to locate the trocar by leveraging information from images. Work in [11] used microscope images to detect the trocar position for vitreoretinal surgery. Its outputs were sensitive to measurement noise giving an average MSE of 226 mm during robotic tests and an average RMSE of 4.62 mm in a constrained and simplified manual experiment. Research in [12] used images from a camera mounted on the robot end-effector to estimate the trocar’s pose for automatic trocar docking. Deep learning requires many training images to learn with accuracy the trocar location. To supplement the training, synthetic images are used, which despite improving the outcome do not entirely capture all the possible appearances of the operating scene.

The method proposed in this paper is suitable for use with a small trocar such as those in vitreoretinal surgery (2.7 mm diameter). The markers will not get occluded by the surgeon/equipment due to them being placed directly on the trocar with the camera being a short distance away, on the tool’s handle. The camera locates the trocar instead of estimating the RCM and assuming its alignment. Furthermore, it does not depend on thousands of training images and functions under different scene conditions, such as blood on the eye or reflection on the sclera. This is due to it depending on well-defined markers and their plurality rather than on scene features. In general, exploring the use of in situ markers for trocar localisation has never been done before.

## Materials and methods

### Previous work

This study used the hardware developed in previous work [4]. The system included an Aurora^®^ EM tracking solution by NDI Inc. (Canada) and a MinnieScope-XS micro-camera from Enable Inc. (USA). The camera was attached to the forceps using a bespoke 3D printed component.

### Trocar localisation

During vitreoretinal surgery, the scene may include artefacts that obfuscate the images captured by the camera, making trocar localisation challenging. Examples include blood covering key features, image specularity due to illumination changes and a dynamic scene with non-rigid tissues.

A promising solution to overcome these challenges are fiducial markers, which present well defined features, allowing for accurate localisation and reliable pose measurements. These properties are essential for an application such as vitreoretinal surgery, which uses instruments of submillimetre diameter and requires submillimetre precision. ArUco markers were chosen due to their consistent performance in detection rate and measurement accuracy for both position and orientation measurements when compared to other marker types [13]. They also present one of the lowest computational cost, making it ideal for any possible future use in embedded platforms.

**Fig. 1:**
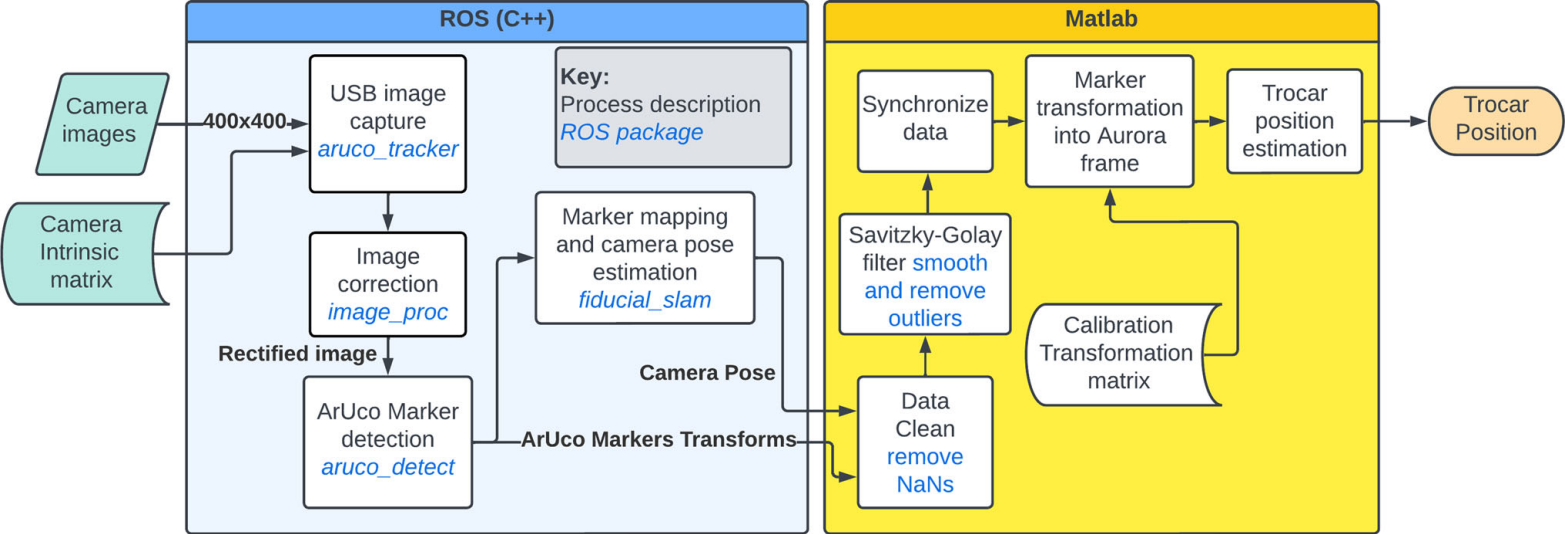
Pipeline showing the processing of camera images to estimate the trocar position

The pipeline used for the trocar localisation is shown in Fig. 1. The camera image size was $400\times 400$ pixels and was calibrated using the ROS $camera\_calibration$ package and a $10\times 7$, 12 mm squared checker board.

The ArUco size used was $3\times 3$ mm with a $4\times 4$ inner square matrix. The size was small enough not be obtrusive, yet large enough to be accurately detected. The smallest pre-defined ArUco dictionary available (DICT_4X4_50) was used, as not many markers were required.

Two ArUco markers were placed on either side of the trocar to locate it in the camera coordinate frame. Knowing the position of the markers, the trocar position was estimated by interpolating the midpoint. A bio-compatible trocar attachment will be created to hold these markers, but this will be the subject of future work. In addition, biocompatible KeyDot^®^ markers by Key Surgical (UK) may substitute the ArUco markers in the future.

### Data acquisition and processing

A Dell G5 15” laptop with an Intel core i7-8750H processor and running Ubuntu 16.04 LTS was used to collect the data. The Kinetic ROS version was used for the data acquisition whilst Matlab R2019b version was used for the data processing. The processing was completed offline for this study. Online real-time processing is part of our future work. The Aurora System Control Unit and the micro-camera interface box were connected via USB to the laptop. The micro-camera light source was set to low intensity to allow the image to be evenly illuminated, avoiding specularities.

The camera pose and marker transforms were recorded using rosbag, which timestamped all the transmitted data messages. Before any data could be used, the data records were reformatted to remove any NaNs produced as a result of markers moving out-of-view of the camera. The inputs were then filtered using the Savitzky–Golay filter to remove any outliers and smooth the signal. Signals were then synchronised and re-sampled at 0.01 s.

**Fig. 2:**
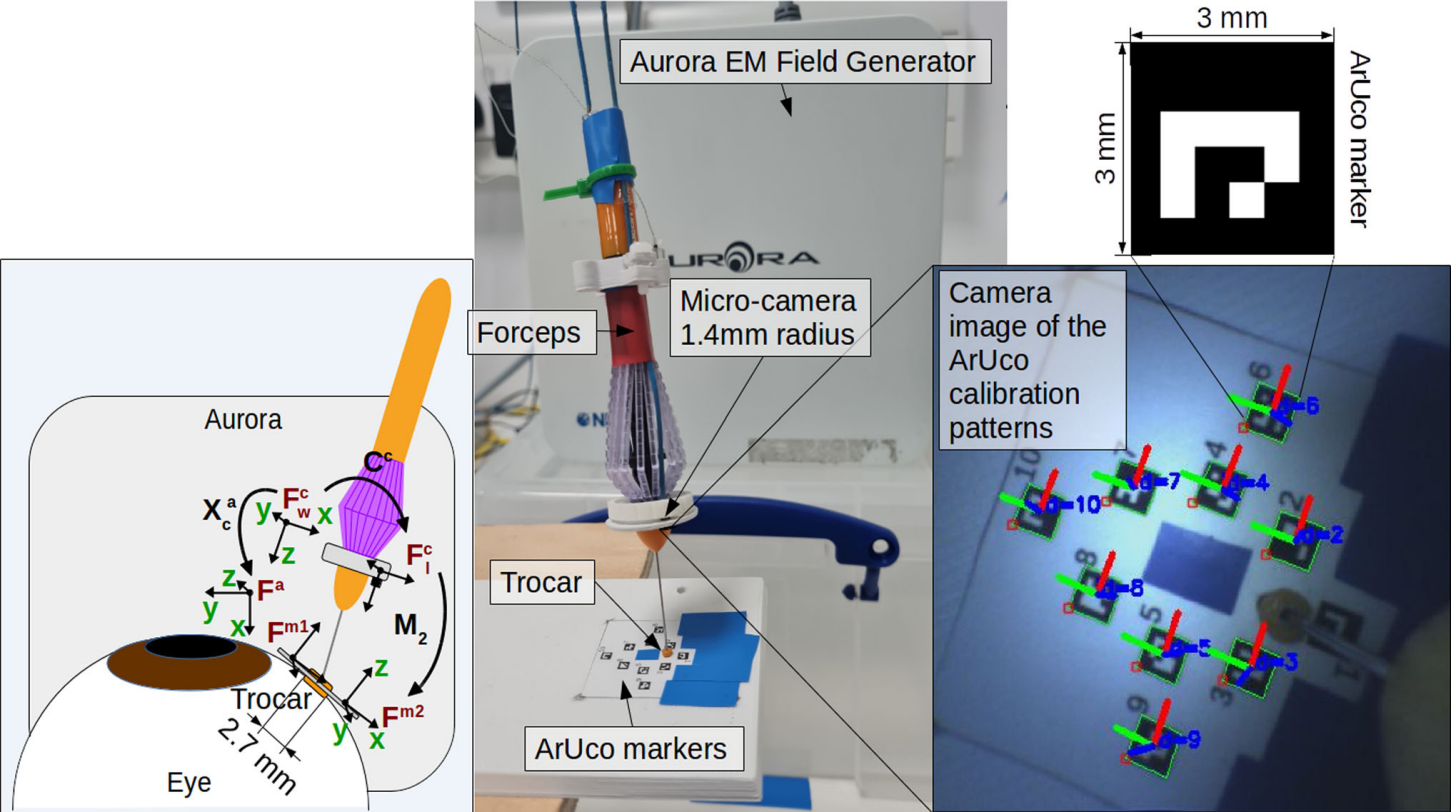
Hardware setup, coordinate frames of the system and ArUco calibration pattern

### Calibration

The Aurora was only used to provide the ground truth for the trocar localisation, having an accuracy RMS of 0.48 mm (position) and ${0.30}^{\circ }$ (orientation). For this reason, the trocar position had to be estimated within the Aurora frame. To find the transformation between the camera and the Aurora frames, registration between both sensors was performed using Horn’s quaternion method [14], which is a common hand-eye calibration technique.

To perform this registration, 9 markers were placed within the camera’s static view and the Aurora’s workspace. The position of each marker was first measured by placing the tip of a standard Aurora 6D 65 mm straight probe (pointer) on it. To ensure consistency of the manual measurements, which were subject to human errors, each pointer test was performed three times at 5 s intervals, with the resulting data for each test merged into one dataset for processing. The markers were then captured using the camera. The data were cleaned to remove any missing records. To remove outliers, the median absolute deviation (MAD) technique was used. Preliminary experimentation showed that any data located above 3.5 scaled MADs from the median for the pointer data, and 2.5 for the camera pose, was considered an outlier and removed. This removed any effect that noise had on the measurements. Following this, for each sensor, the centroid of the measurements was calculated using their median. This way, the resulting value for the position of the marker would consider any skewing of the data and therefore make the resulting calibration less sensitive to measurement noise.

The positions of the markers as recorded by both the EM and the camera sensor, in their respective coordinate frames, were input into Horn’s function, which estimated the transformation matrix between them. The different coordinate frames of the system are shown in Fig. 2. To transform the markers into the Aurora frame, first they had to be transformed into the camera world frame ($F^\textrm{c}_\textrm{w}$). To achieve this, the marker transform in the camera’s local frame, *M*, was multiplied by the camera’s homogeneous transformation, $C^c$.

1 $$\begin{aligned} M^\textrm{c} = C^c \cdot M \end{aligned}$$

Before transforming $M^\textrm{c}$ into the Aurora frame, the *z*-axis position was reversed. This was due to the camera world frame being left-handed, whilst the Aurora frame was right-handed.

2 $$\begin{aligned} M^\textrm{c}(3,4) = -M^\textrm{c}(3,4) \end{aligned}$$

The transformation into the Aurora frame was then carried out by multiplying $M^\textrm{c}$ by the calibration transformation matrix, $X^\textrm{a}_c$.

3 $$\begin{aligned} M^\textrm{a} = X^\textrm{a}_c \cdot M^\textrm{c} \end{aligned}$$

## Experiments and results

### Set-up

For the experiments, a bespoke 3D printed platform was created (Fig. 2). Metal was avoided in the setup to prevent interference with the Aurora’s magnetic field. A vitrectomy 23-gauge trocar was placed on the platform, whilst the ArUco markers were stuck onto the surface around the trocar. This allowed for both the calibration and testing steps to be performed consecutively.

### Camera-Aurora registration performance experiments

These experiments investigated the accuracy and consistency of the marker transformation process into the Aurora frame. Determining these metrics is critical, as inaccuracies produced by this process affect trocar localisation. Accuracy of the trocar estimation was also tested. The error metrics used were the mean bias error (MBE) to analyse the bias of the results, the median absolute deviation (MAD) to analyse the variability and root mean squared error (RMSE) the main error metric that penalises large errors. Three tests for each of the three experiments were carried out: static experiment whilst in the calibration position, static experiment once the forceps had been rotated, and motion experiment whilst pivoting for 60 s about the trocar.

#### Experiment A: static in calibration pose

The 9 calibration markers were used to validate calibration and measure its accuracy before the forceps was moved. The marker positions in the Aurora frame were compared against the ground truth. Table 1 presents the results.

**Table 1:** Averaged localisation errors for 9 ArUco markers over three tests whilst static

All in mm	MBE	MAD	RMSE
*x*	$-$ 0.04	0.66	0.66
*y*	$-$ 0.02	0.26	0.26
*z*	0.00	0.30	0.31
Eucl. Dist.	0.83	0.83	0.84

**Table 2:** Localisation errors for 3 ArUco markers over 3 tests, whilst static after moving the forceps from the initial calibration pose

RMSE (mm)	Fid 1	Fid 2	Fid 3	Average (MBE $\Vert$ MAD $\Vert$ RMSE)
*x*	1.47	0.54	1.26	$-$ 0.71 $\Vert$ 1.05 $\Vert$ 1.09
*y*	0.72	0.20	0.37	$-$ 0.09 $\Vert$ 0.39 $\Vert$ 0.43
*z*	0.61	0.17	0.28	$-$ 0.12 $\Vert$ 0.29 $\Vert$ 0.36
Eucl. Dist.	1.76	0.61	1.36	1.21 $\Vert$ 1.21 $\Vert$ 1.24

**Table 3:** Localisation errors for 3 ArUco markers over 3 tests, whilst pivoting for 60 s

RMSE (mm)	Fid 1	Fid 2	Fid 3	Average (MBE $\Vert$ MAD $\Vert$ RMSE)
*x*	1.39	0.80	2.47	$-$ 1.04 $\Vert$ 1.25 $\Vert$ 1.55
*y*	0.83	0.62	0.62	$-$ 0.21 $\Vert$ 0.39 $\Vert$ 0.69
*z*	0.58	0.45	0.55	0.02 $\Vert$ 0.33 $\Vert$ 0.53
Eucl. Dist.	1.73	1.12	2.61	1.59 $\Vert$ 1.46 $\Vert$ 1.82

#### Experiment B: static after motion

To assess the consistency of the transformation process, the forceps was rotated by ${180}^{\circ }$ to change the camera pose from the initial calibration position. Only two previously tracked markers were in view (ID 2 and ID 3) as these were the ones used for trocar localisation. Additionally, a new marker (ID 1) was placed in view to validate the robustness of the transformation against a new point. Tests lasting for 5 s each were carried out, with the resulting data filtered to remove outliers. The results are shown in Table 2.

#### Experiment C: pivoting motion

**Fig. 3:**
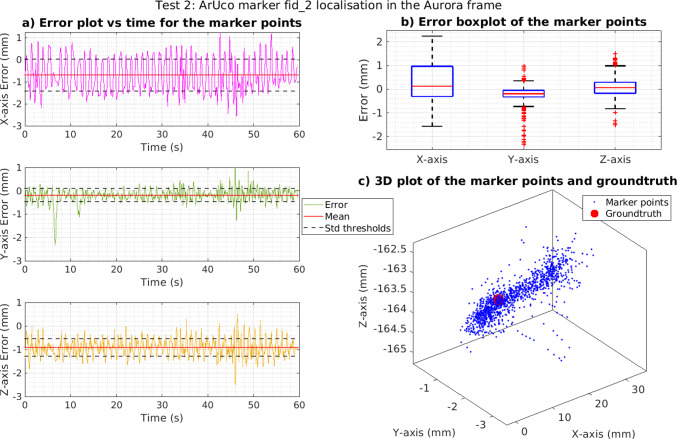
60-s marker localisation pivoting test results. **a** Error versus time for each axis. **b** Boxplot error for each axis, with the median, 25th and 75th percentiles and outliers as red crosses. **c** 3D plot of all the estimated trocar locations, with the red point being ground truth

To assess the performance of the transformation process during camera motion, the forceps was pivoted about the trocar for 60 s at $\pm \,45^{\circ }$ from vertical position. During this period, the camera always had at least one of the three markers in view. Pivoting was chosen as this would be the normal kind of motion performed during surgery. The test results are outlined in Table 3. Figure 3 presents an example of the results for a marker in one of the tests.

### Trocar localisation performance experiments

**Fig. 4:**
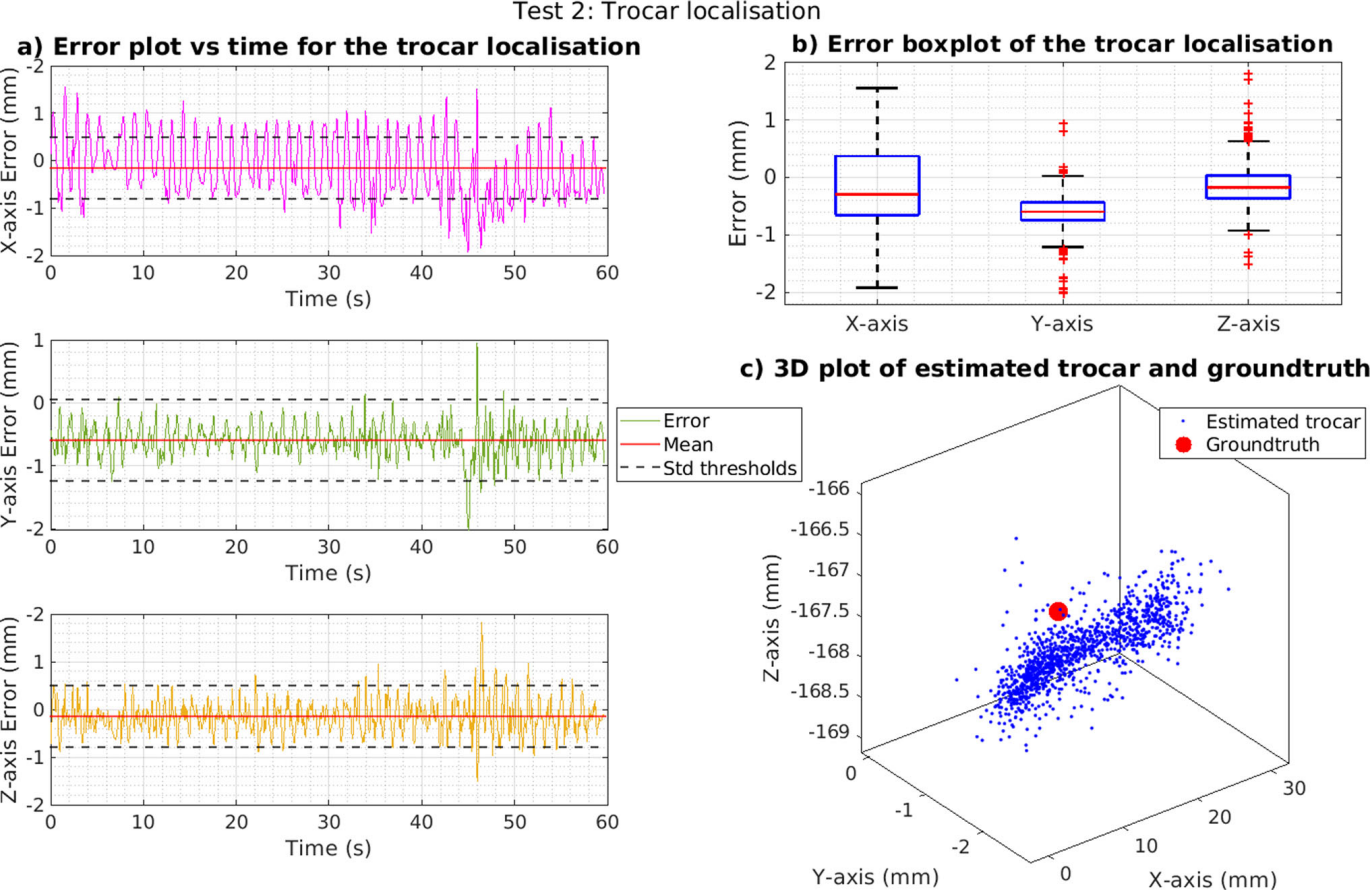
60-s trocar localisation pivoting test results. **a** Error versus time for each axis. **b** Boxplot error for each axis, with the median, 25th and 75th percentiles and outliers as red crosses. **c** 3D plot of all the estimated trocar locations, with the red point being ground truth

To assess the performance of the trocar localisation, the data collected for experiments B and C were used to estimate the trocar position in the Aurora frame. The results are shown in Table 4 for the static tests and Table 5 for the pivoting tests. Figure 4 presents the results from one of the pivoting tests.

## Discussion

### Registration process validation

Results from the static tests proved a good fit of the calibration transformation matrix to the data. An overall Euclidean distance RMSE of 0.84 mm and MAD of 0.83 mm was achieved, showing low variability and good accuracy.

For experiment B, when the forceps was rotated, both the accuracy and consistency of the transformations decreased. This was caused by increased measurement noise and errors in the camera pose. It was observed that, once moved, the camera’s *x*-axis static position had a 6 mm range, which was the largest source of error. For experiment A, the camera’s *x*-axis position had a 2 mm range. Due to most of the points being clustered around a small area, this range decreased to 0.6 mm after outlier rejection. However, for experiment B the outlier rejection was not successful as the camera’s positions were evenly spread out along the 6 mm range. This meant that all data points were considered when calculating the median. The effect of the camera *x*-axis position’s inconsistencies can be seen throughout all the tests. The marker’s *x*-axis had the largest error bias, being at an average of $-0.71$ mm from the ground truth.

The pivoting test results showed greater errors and variance compared to static tests, which were attributed to the increased measurement and process noise during motion. This equated to about a 0.58 mm increase for the Euclidean distance RMSE, bringing the value to 1.82 mm.

### Trocar localisation

The trocar localisation results follow the same pattern as previous experiments. The static tests results show a good consistency between tests, but small biases in the measurements, especially the *x*-axis. The pivoting experiment showed an increase in error when compared to the static tests. Upon further investigation, it was found that marker ID 3, used to calculate the trocar position, was out of view intermittently during the test or was not detected due to changes in the camera’s view and lighting conditions. This is shown by it having the largest error of all the markers. As a consequence, the interpolated trocar positions would incorporate inaccuracies and inconsistencies within the test. To improve the robustness of the trocar localisation algorithm, it is suggested that 3 or 4 markers are placed around the trocar. This would both increase the localisation’s accuracy and ensure that at least two markers are in view at all times for the trocar position interpolation. A balance has to be struck between simplifying the trocar attachment and increasing localisation performance. Careful consideration will be given to its design to ensure that the eye is not crowded neither the lens occluded, which surgeons look through to view the retina during surgery. Some of the errors could have been introduced by the marker’s measurement error, which would propagate to the trocar estimation. Using a Kalman filter to find the optimum trocar position over time considering the measurement errors might improve this.

**Table 4:** Trocar localisation errors across 3 tests, whilst static

RMSE (mm)	Test 1	Test 2	Test 3	Average (MBE $\Vert$ MAD $\Vert$ RMSE)
*x*	0.50	0.47	0.72	0.28 $\Vert$ 0.42 $\Vert$ 0.56
*y*	0.54	0.38	0.40	$-$ 0.41 $\Vert$ 0.40 $\Vert$ 0.44
*z*	0.36	0.26	0.31	$-$ 0.23 $\Vert$ 0.26 $\Vert$ 0.31
Eucl. Dist.	0.82	0.65	0.88	0.75 $\Vert$ 0.68 $\Vert$ 0.78

**Table 5:** Trocar localisation errors across 3 tests, whilst pivoting for 60 s

RMSE (mm)	Test 1	Test 2	Test 3	Average (MBE $\Vert$ MAD $\Vert$ RMSE)
*x*	0.87	0.67	0.76	$-$ 0.37 $\Vert$ 0.60 $\Vert$ 0.77
*y*	1.12	0.65	0.82	$-$ 0.65 $\Vert$ 0.62 $\Vert$ 0.87
*z*	0.54	0.38	0.44	$-$ 0.15 $\Vert$ 0.27 $\Vert$ 0.46
Eucl. Dist.	1.52	1.01	1.21	1.08 $\Vert$ 0.99 $\Vert$ 1.24

For trocar localisation, the axis displaying the depth (*x*-axis for the Aurora frame) is the most important one. When the algorithm is used as feedback for the control of the RCM, if the depth value is incorrect, the eye will be pushed or pulled by the pivoting tool. In the static tests, the *x*-axis has the greatest error, $\times 1.2$ and $\times 1.8$ more than *y*/*z*-axis, whilst in the pivoting tests it has the second greatest ($\times 1.6$ more than *z*-axis but $\times 0.8$ of the *y*-axis error). A potential improvement could be the use of two cameras for stereo-vision, which would enhance depth perception and reduce the number of markers required around the trocar. Alternatively, reducing the camera’s field of view would increase both the pixels occupied by the marker and the capability for depth resolution. During the pivoting tests, the *y*-axis marginally had the largest error ($\times 1.1$ more than the *x*-axis). The pivoting motion was primarily done along this axis, thus displaying the largest changes in position. The fast position changes and the increased error difference between detected markers could account for the error. All the aforementioned solutions should also ameliorate this, reducing the error and improving the consistency of measurements.

The proposed technique attained an improvement over previous research [11], which gave an RMSE of 4.62 mm. Comparatively our static and more complex pivoting tests had a RMSE of 0.78 mm and 1.24 mm, respectively.

## Conclusion

This paper presents a trocar localisation method for use in vitreoretinal procedures, particularly for robot-assisted surgery. A single micro-camera detected two 3 mm ArUco markers at either side of the trocar, which were used to estimate its position. A reasonable accuracy between tests was achieved, supporting that this method is a viable way to achieve trocar localisation. The optimum RMSE for this application would be less than 1.4 mm, corresponding to the trocar’s radius. Future work will investigate ways to minimise the localisation error and stay consistently within this margin. This includes increasing the number of markers and adding an extra camera for stereo-vision. Deep learning could be used to estimate the pose of the ArUco markers, improving its robustness against motion blur, poor lighting or semi-occlusion.
